# Clinical outcomes with a new design in multifocal intraocular lens: a pilot study

**DOI:** 10.1186/s40662-020-00205-3

**Published:** 2020-07-18

**Authors:** Jorge L. Alió, Pilar Yébana, Mario Cantó, Ana B. Plaza, Alfredo Vega, Jorge L. Alió del Barrio, Francisco Lugo

**Affiliations:** 1 Vissum Corporation, Avda de Denia s/n, Edificio Vissum, 03016 Alicante, Spain; 2 Division of Ophthalmology, Universidad Miguel Hernández, Alicante, Spain

**Keywords:** Multifocal intraocular lens, Near vision, Photic phenomena, Continuous transitional focus optic

## Abstract

**Purpose:**

To evaluate the clinical and visual outcomes, quality of near vision and the influence of photic phenomena in patients bilaterally implanted with a new Precizon Presbyopic multifocal intraocular lens (IOL).

**Methods:**

In this prospective consecutive case series, 20 eyes of 10 patients were included (mean age 63.80 ± 12.55 years). Uncorrected and corrected visual acuity (far, intermediate and near), subjective refraction, binocular defocus curve, contrast sensitivity (CSV-1000) and quality of vision and satisfaction questionnaires were measured. The follow-up was 12 months after surgery.

**Results:**

At 12 months after surgery, uncorrected distance visual acuity (UDVA) improved with surgery (*p* = 0.001) with a value of 0.08 ± 0.08 logMAR. Uncorrected near visual acuity (UNVA) was 0.22 ± 0.12 logMAR and distance corrected near visual acuity (DCNVA) was 0.16 ± 0.13 logMAR. Intermediate distance visual acuity (UIVA) was 0.22 ± 0.10 logMAR. Contrast sensitivity outcomes were similar to normal population in photopic conditions and slightly reduced in mesopic conditions of lighting. Defocus curve showed that this multifocal IOL was able to provide a visual acuity (VA) equal or better to 0.16 logMAR between defocus levels of + 1.00 to − 2.50 D. Good patient satisfaction was obtained in quality of vision and satisfaction questionnaires outcomes.

**Conclusions:**

The Precizon Presbyopic NVA IOL (OPHTEC BV) provides good visual outcomes. This multifocal IOL provides a high percentage of spectacle independence due to good VA at far, intermediate and near distances and satisfactory contrast sensitivity. High patient satisfaction was observed in quality of vision and satisfaction questionnaires with a low percentage of patients manifesting photic phenomena.

## Introduction

Nowadays, the demand for multifocal intraocular lenses (IOLs) has increased which can be attributed to the increase of younger patients that demand a solution for their presbyopia and look for spectacle independence. There are a lot of IOLs and designs to provide an excellent visual performance on various distances of sight. These IOLs look for satisfactory visual acuity (VA) and a suited quality of vision for near, intermediate and far vision [1–4].

**Fig. 1:**
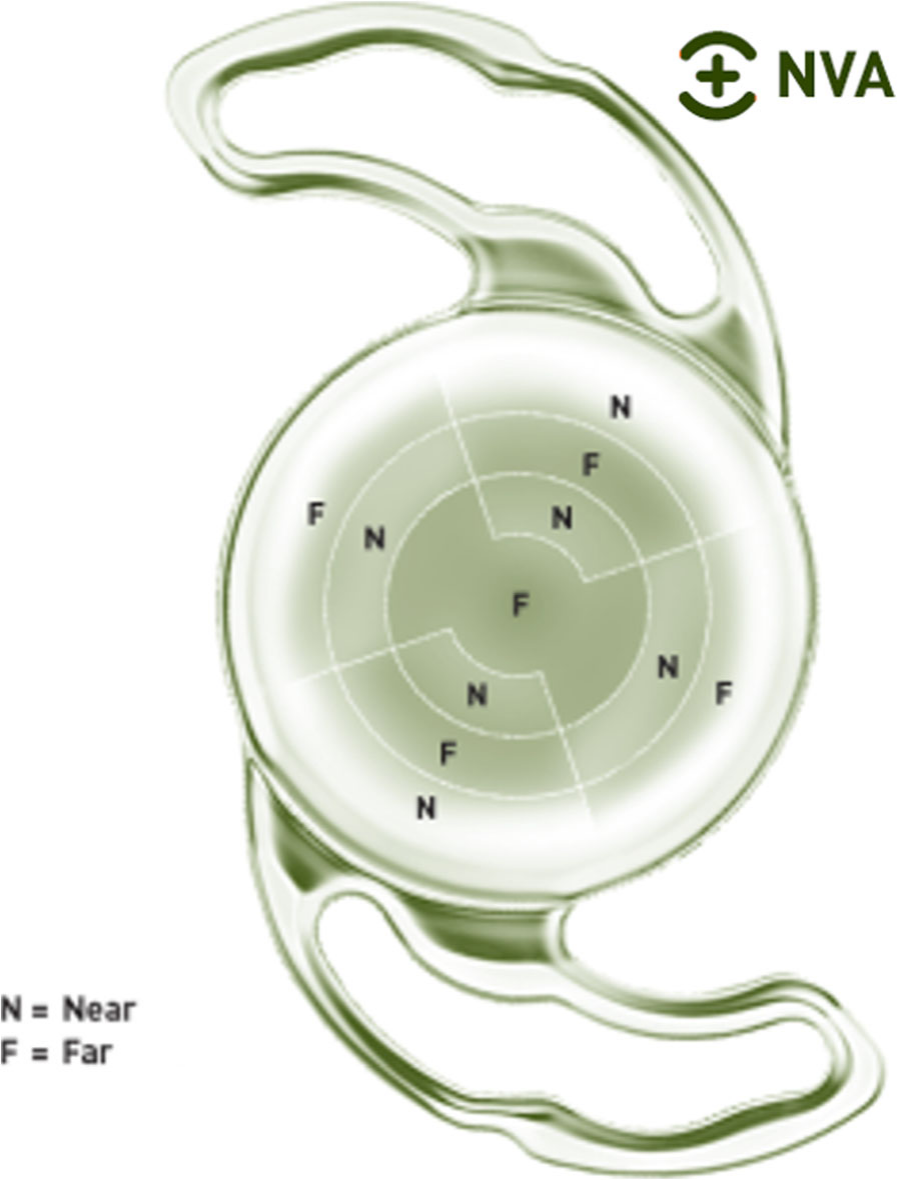
Precizon Presbyopic multifocal IOL. Design pupil-independent aspherical segmented refractive optic

The innovation of this Precizon Presbyopic NVA (OPHTEC BV) multifocal intraocular lens (IOL) lies in its new design, an aspherical segmented refractive optic, which conforms a continuous transitional focus (CTF) optic (Fig. 1). This is an optic with an anterior surface with multiple segments for far and near. A smooth transition from far to near is achieved between the segment. This transition offers a constant defocus between the two sharp focal points, delivering a good intermediate vision. Due to the design, patients benefit from good vision at difference distances independently from the size of the pupil diameter. The use of segments instead of concentric rings should provide better optical quality and reduce photic phenomena.

The aim of this study is to evaluate visual outcomes, quality of near vision and the influence of light distortion in patient bilaterally implanted with this multifocal Precizon Presbyopic IOL.

## Methods

### Study design

This is a monocentric clinical interim pilot study reporting data at 12 months, from one investigation site. This study is a pilot prospective consecutive non-comparative case series.

### Patients

Twenty eyes of 10 patients were bilaterally implanted with the Precizon Presbyopic multifocal IOL. Mean age of the patients was 63.80 ± 12.55 years (range: 42 to 79 years). All patients agreed to participate and signed an informed consent. The study adhered to the tenets of the Declaration of Helsinki and was approved by the local ethical committee (Ethics Committee for Drug Research of Cádiz approved this study as an audit study and gave it the following reference number AP01000740).

### Intraocular Lens

The Precizon Presbyopic IOL NVA model 570 (OPTHEC BV) is a one-piece IOL made of a hybrid material hydrophilic/hydrophobic acrylic material with ultraviolet filtering HEMA/EOEMA copolymer, with a refractive index of 1.46. The size of the clear optic diameter is 4.4 mm, with an overall diameter of 12.5 mm.

A multi-zonal design allows the lens to maintain the light distribution and exposure on the foci theoretically regardless of the tilt or decentering of the lens. This IOL provides the ability for a transition in focus between 11 distinct segments (five for distance and six for near vision) with the central segment dedicated for distance vision (Fig. 1). The rotated segments have a width of 0.75 mm and these segments are distributed in such a way that decentration or pupil size has a minimal effect on the ratio between near and far correction [5].

**Fig. 2:**
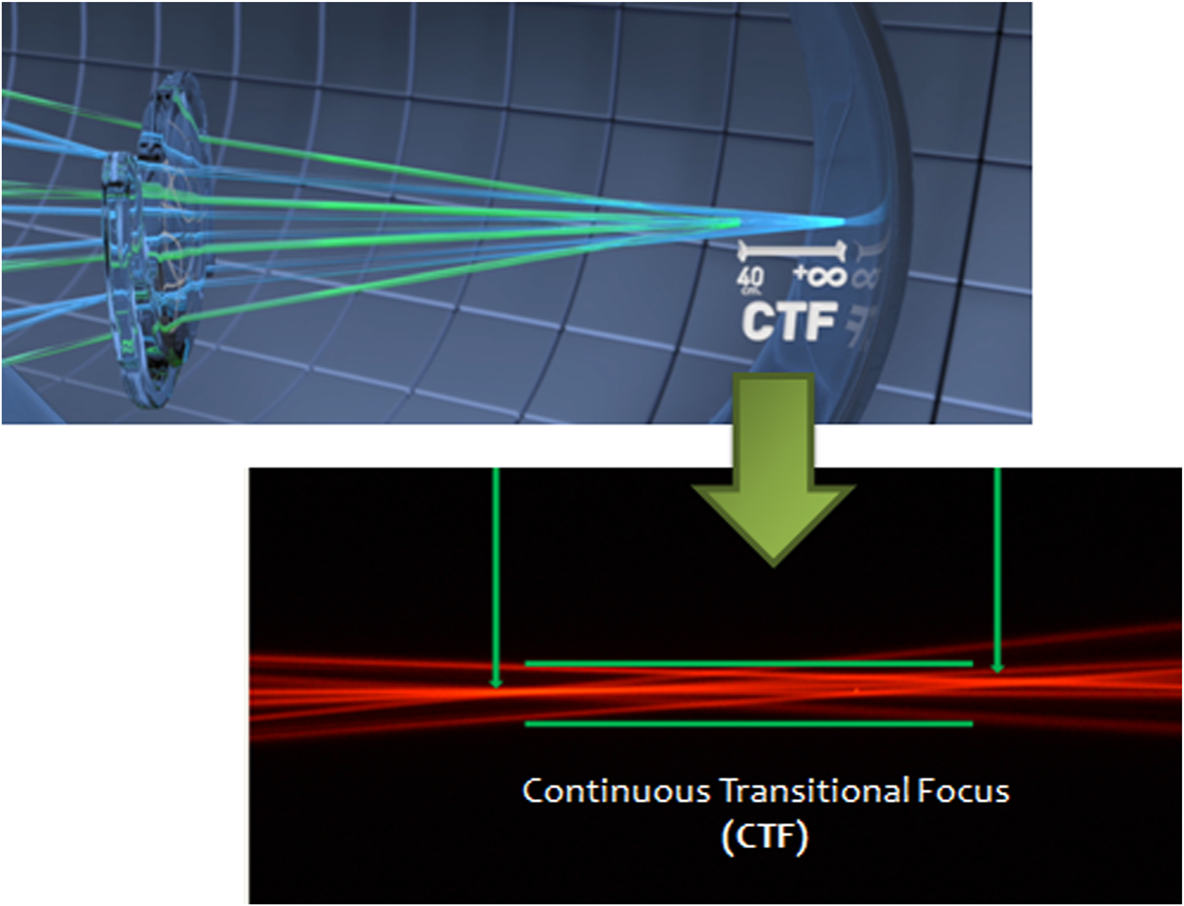
Continuous Transitional Focus (CTF) optic offers a constant defocus between the two sharp focal points

The IOL optic is designated as CTF and is divided into three concentric sectors: the central sector, of higher diameter, is dedicated to distance correction; two peripheral sectors present a bimodal (50–50%) distribution of distance and near correction, and this distribution changes along four segments in each sector. This new optic design with an anterior surface with multiple segments for far and near achieves a soft transition from far to near focus. This transition theoretically offers a constant progressive focus between the two sharp focal points to facilitate a sharp image on the retina delivering a good intermediate vision (Fig. 2) [6].

This IOL has a range optic power between + 1.0 and + 35.0 D (0.5 D increments) and has an addition of + 2.75 D for near focus.

### Inclusion and exclusion criteria

The inclusion criteria of this study were candidates with early cataract and presbyopia looking for spectacle independence (cataract or clear lens extraction patients) who qualify for bilateral implantation and patients wishing to be spectacle independent. Exclusion criteria were the presence of any other ocular pathology which could compromise visual outcomes (amblyopia, anterior segment comorbidities, glaucoma, retinal disorders or neuro-ophthalmic disease) and preoperative astigmatism higher than 1.0 D measured with optical biometer or corneal topography.

### Preoperative and postoperative examination

All patients were evaluated preoperatively and for each operated eye at day 1, week 1 and months 1, 3 and 12 after surgery.

All measurements were performed by two independent optometrists certified in Good Clinical Practice (ABP and PY) following the same investigational protocol according standard procedures.

The preoperative examination contained an extensive evaluation including: distance visual acuity (DVA) at 4 m with early treatment diabetic retinopathy study (ETDRS) logMAR charts, subjective refraction, contrast sensitivity in photopic and mesopic conditions with and without glare in binocular vision (CSV-1000, Vector Vision), biometry (IOL Master, Zeiss), pupillometry (Sirius, Costruzione Strumenti Oftalmici), slit-lamp examination, topography (Sirius, Costruzione Strumenti Oftalmici) and quality of vision questionnaire.

Postoperatively, patients were evaluated during the follow-up at 1 day, 1 month, 3 months and 12 months after surgery according the same protocol which included: DVA (ETDRS at 4 m), near visual acuity (NVA) (ETDRS reading chart calibrated at 40 cm), intermediate visual acuity (IVA) (ETDRS reading chart calibrated at 80 cm), subjective refraction, contrast sensitivity (in the same conditions to preoperative evaluation), defocus curve in binocular vision, slit-lamp examination, quality of vision [7] and satisfaction questionnaire (a questionnaire adapted for this study was used).

Defocus curve was performed in binocular conditions with the postoperative manifest refraction corrected following the protocol previously published [8–10]. The defocus curve was obtained using ETDRS charts at 4 m. Negative lenses were added in 0.50 D steps and the VA was recorded for each type of defocus level. Afterwards, the same procedure was repeated using positive lenses. The range of defocus curve evaluated was from − 5.00 to + 1.50 D. In the defocus curve, 0.00 D of defocus corresponds to far vision, − 1.50 D of defocus corresponds to intermediate vision and − 2.50 D of defocus to near vision.

Contrast sensitivity vision was performed in binocular conditions at a distance of 2.5 m for 4 frequencies in cycles per degree (cpd) (A: 3 cpd, B: 6 cpd, C: 12 cpd and D: 18 cpd) and patients were tested with the postoperative manifest refraction corrected. Four measurements have been performed for each patient, in photopic and mesopic conditions with and without glare. To measure contrast sensitivity in mesopic light condition we used specially designed mesopic (neutral density) filters to reduce the testing light level. The CSV-1000 test provides a glare option, this test incorporates a standardized halogen glare test so it can be used under glare conditions.

### Statistical analysis

The statistical analysis was performed with the SPSS software for Windows (IBM SPSS Statistics, version 22.0). Due to small sample size (*n* < 30), our variables were considered non-normal, and thus we used non-parametric tests for data analysis.

For the continuous variables observed, the Wilcoxon test was used to assess the difference between preoperative and postoperative outcomes. Differences were considered statistically significant when the *P* value was less than 0.05.

## Results

A total of 20 eyes of 10 patients (70% cataract; 30% clear lens extraction) were examined 12 months postoperatively.

### Visual acuity and refractive status

Table 1 shows the monocular postoperative refraction and VA in logMAR results 3 and 12 months postoperatively. Uncorrected distance visual acuity (UDVA) improved with the surgery at 3 and 12 months (*p* < 0.001), it even had a significant improvement between the third and twelfth month (*p* = 0.015). Corrected distance visual acuity (CDVA) improved significantly after surgery (*p* < 0.05) and remained stable over time (*p* = 0.074). The outcomes at 12 months of uncorrected near visual acuity (UNVA) at 40 cm and distance corrected near visual acuity (DCNVA) at 4 m was 0.22 ± 0.12 logMAR and 0.16 ± 0.13 logMAR respectively, remaining stable over time between postoperative visits (*p* > 0.05). At 3 months, corrected near visual acuity (CNVA) was 0.08 ± 0.05 logMAR, and for intermediate vision the results showed an uncorrected intermediate visual acuity (UIVA) of 0.22 ± 0.10 logMAR.

**Table 1:** Postoperative monocular refractive results and visual acuities at 3 and 12 months

	Preoperative Visit	Postoperative Visit 3 Months	Postoperative Visit 12 Months	*p*-value	*p*-value	*p*-value
Refractive status	Mean ± SD	Mean ± SD	Mean ± SD	Pre-3 M	Pre-12 M	3 M–12 M
Sphere (D)	0.77 ± 1.87	0.58 ± 0.41	0.62 ± 0.48	0.641	0.675	0.762
Cylinder (D)	− 0.67 ± 0.69	−0.46 ± 0.42	−0.49 ± 0.39	0.209	0.256	0.832
SE (D)	0.43 ± 2.05	0.36 ± 0.42	0.38 ± 0.48	0.857	0.888	0.838
Visual Acuity (logMAR)						
UDVA	0.51 ± 0.30	0.13 ± 0.09	0.08 ± 0.08	< 0.001*	< 0.001*	0.015*
CDVA	0.13 ± 0.22	0.02 ± 0.06	− 0.01 ± 0.05	0.033*	0.012*	0.074
UNVA	–	0.22 ± 0.11	0.22 ± 0.12	–	–	0.868
DCNVA	–	0.18 ± 0.10	0.16 ± 0.13	–	–	0.242
CNVA	–	0.08 ± 0.05	–	–	–	–
UIVA	–	0.22 ± 0.10	–	–	–	–

*SE=* Spherical equivalent, *D=* Diopters, *M=* Months, *UDVA=* Uncorrected distance visual acuity, *CDVA=* Corrected distance visual acuity, *UNVA=* Uncorrected near visual acuity, *DCNVA=* Distance corrected near visual acuity, *CNVA=* Corrected near visual acuity, *UIVA=* Uncorrected intermediate visual acuity

Table 2 shows the binocular VA and refractive results at 3 and 12 months after surgery. Binocularly, the outcomes of this IOL shows an improvement in UDVA after surgery at 3 and 12 months (*p* < 0.05), they even had a significant improvement between the third and twelfth month (*p* = 0.040). The uncorrected visual acuity outcomes of distance and near at 12 months after surgery were − 0.01 ± 0.05 logMAR and 0.13 ± 0.12 logMAR, respectively. Intermediate vision at 3 months was 0.16 ± 0.11 logMAR.

**Table 2:** Postoperative binocular visual acuities at 3 and 12 months

	Preoperative Visit	Postoperative Visit 3 Months	Postoperative Visit 12 Months	*p*-value	*p*-value	*p*-value
Visual Acuity (logMAR)	Mean ± SD	Mean ± SD	Mean ± SD	Pre-3 M	Pre-12 M	3 M–12 M
UDVA	0.34 ± 0.26	0.04 ± 0.04	−0.01 ± 0.05	0.007*	0.002*	0.040*
CDVA	0.01 ± 0.15	−0.03 ± 0.04	−0.11 ± 0.13	0.510	0.057	0.081
UNVA	–	0.15 ± 0.13	0.13 ± 0.12	–	–	0.500
DCNVA	–	0.15 ± 0.14	0.08 ± 0.11	–	–	0.064
CNVA	–	0.08 ± 0.05	–	–	–	–
UIVA	–	0.16 ± 0.11	–	–	–	–

*UDVA=* Uncorrected distance visual acuity, *CDVA=* Corrected distance visual acuity, *UNVA=* Uncorrected near visual acuity, *DCNVA=* Distance corrected near visual acuity, *CNVA=* Corrected near visual acuity, *UIVA=* Uncorrected intermediate visual acuity, *M=* Months

**Fig. 3:**
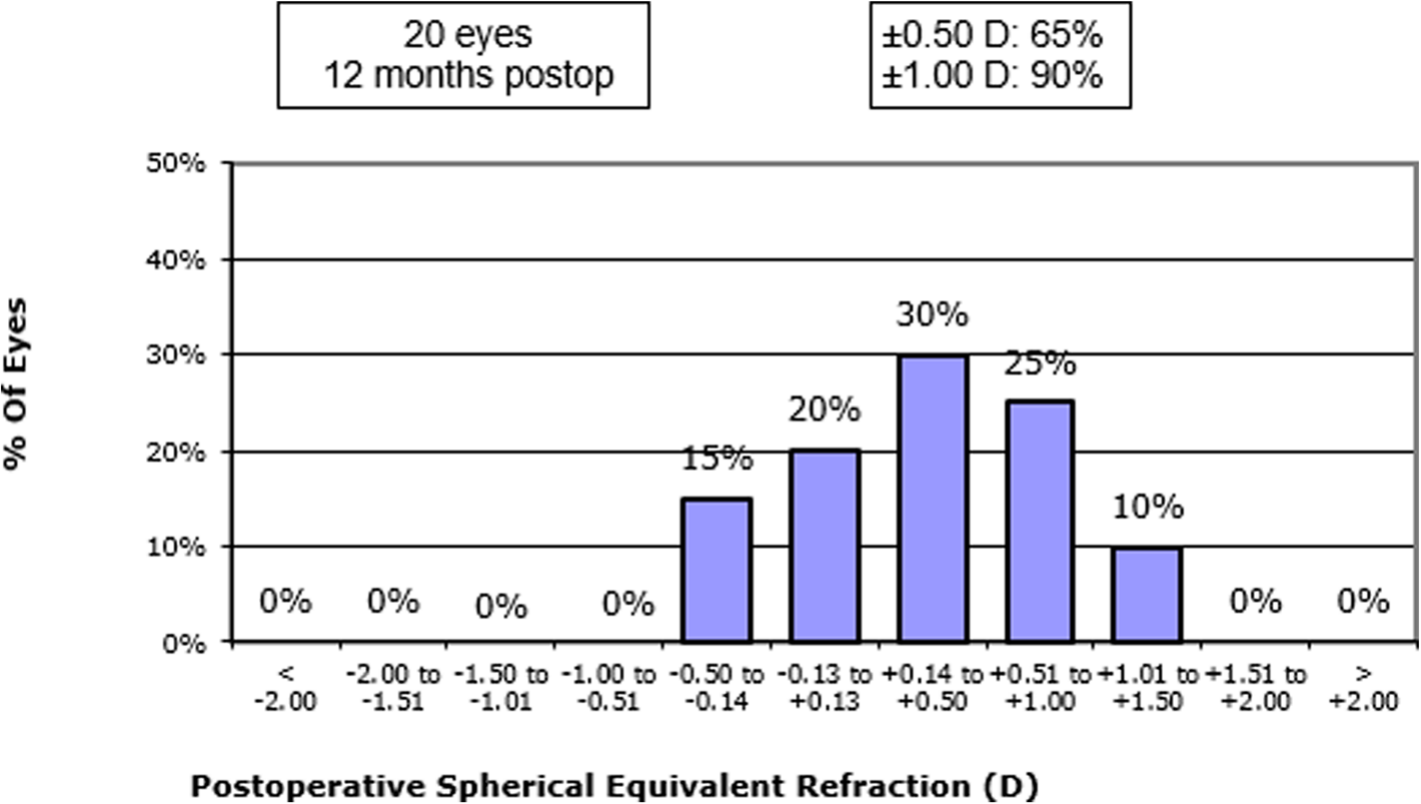
Distribution of the postoperative spherical equivalent at 12 months

Figure 3 shows the distribution of the postoperative spherical equivalent for all eyes at 12 months, 65% of eyes obtained a spherical equivalent between ±0.50 D and 90% of eyes between ±1.00 D.

### Defocus curve

**Fig. 4:**
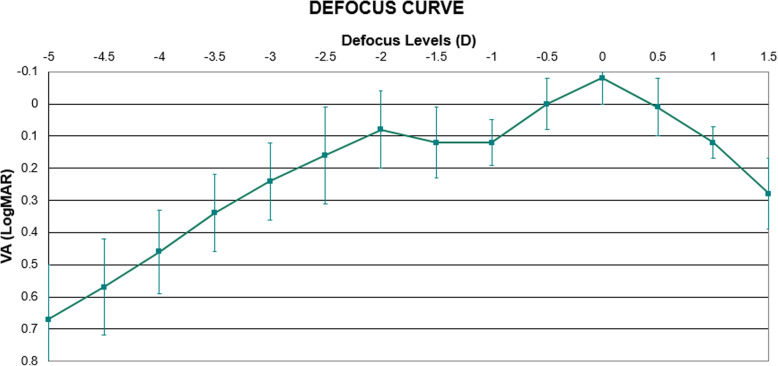
Binocular defocus curve 12 months postoperatively

The binocular defocus curve is shown in Fig. 4. At 12 months, the maximum value of VA obtained was − 0.08 ± 0.08 logMAR corresponding to the far defocus (0.00 D). A second peak was found at − 2.00 D with a VA of 0.08 ± 0.12 logMAR. Corresponding to near defocus (− 2.50 D) the VA obtained was 0.16 ± 0.15 logMAR while for intermediate vision (− 1.50 D) the VA was 0.12 ± 0.11 logMAR. As shown, this multifocal IOL was able to provide a VA equal or better to 0.16 LogMAR between defocus levels of + 1.00 to − 2.50D.

### Contrast sensitivity

**Fig. 5:**
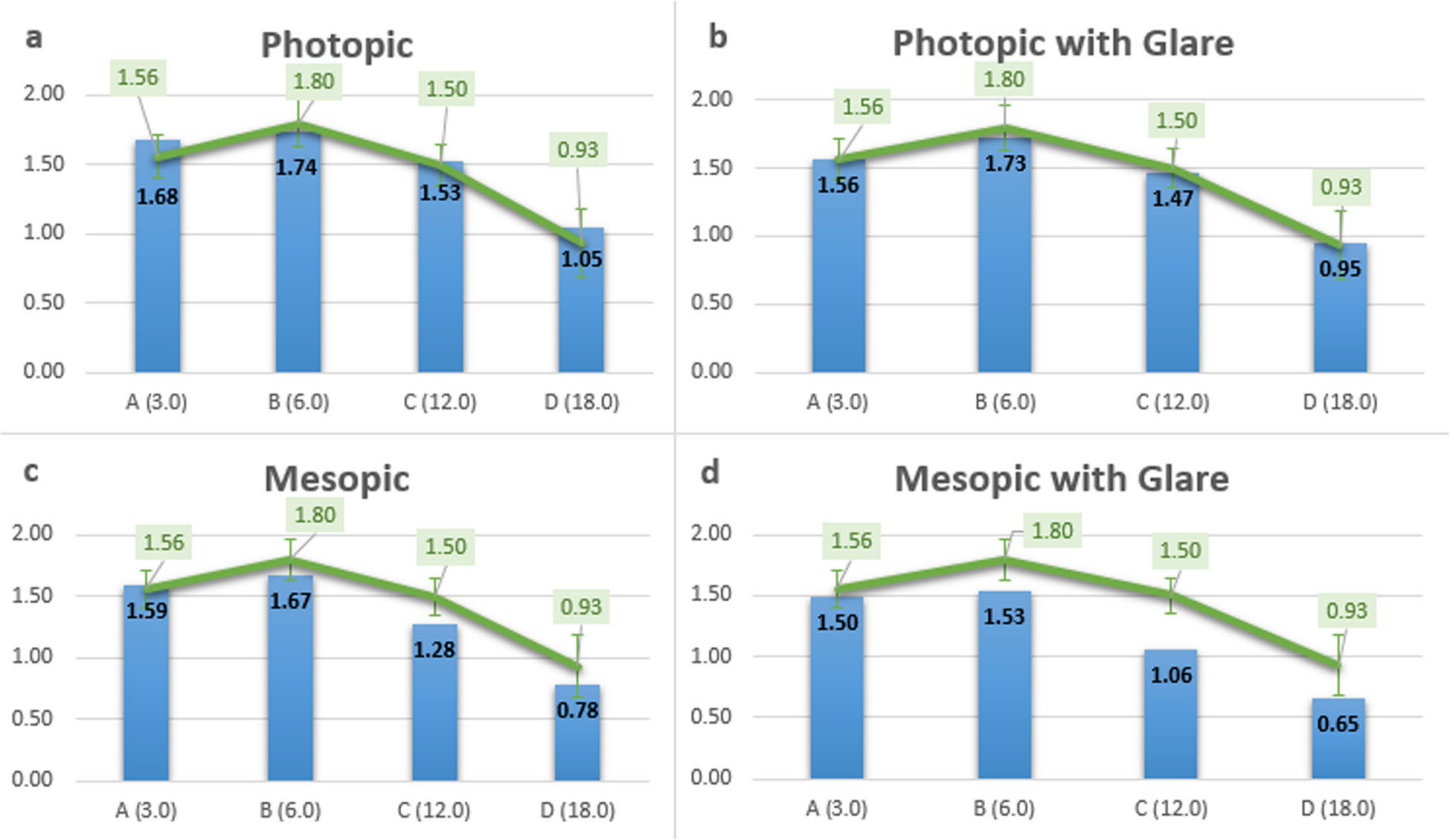
Contrast sensitivity outcomes 12 months postoperatively. **a**, Contrast sensitivity values (in log units) in photopic conditions. **b**, Contrast sensitivity values in photopic conditions with glare. **c**, Contrast sensitivity values in mesopic conditions. **d**, Contrast sensitivity values in mesopic conditions with glare. Green line shows the population norms

The outcomes of contrast sensitivity in photopic and mesopic conditions and measurement with and without glare in binocular vision at 3 months are shown in Fig. 5. This multifocal IOL provides contrast sensitivity values (in log units) similar to those observed in normal population, for an age group between 50 and 75 years of age (Table 3), in photopic lighting conditions (A: 1.68, B: 1.74, C: 1.53, D: 1.05) and slightly reduced in mesopic lighting conditions (A: 1.59, B: 1.68, C: 1.28, D: 0.78).

**Table 3:** Population norms in contrast sensitivity (CSV-1000 test) for an age group between 50 and 75 years of age

	A (3.0)	B (6.0)	C (12.0)	D (18.0)
**Log Values**	1.56	1.80	1.50	0.93
**SD**	0.15	0.17	0.15	0.25

### Questionnaires

#### Quality of vision questionnaire

Table 4 shows the subjective quality of vision reported by patients at 3 and 12 months postoperatively. At 12 months, 70% of patients reported that they “never” observed glare and 30% of patients said that they had seen glares “occasionally”. With respect to halos, 40% of patients reported that they “never” observed halos, 40% “occasionally”, 20% of patients reported that they “often” seen halos, and 0% of patients reported “usually” seeing halos.

**Table 4:** Outcomes of quality of vision questionnaire (3 and 12 months postop)

		3 months	12 months
**1.** How often do you perceive **glares**?	Never	60%	70%
	Occasionally	20%	30%
	Often	10%	0%
	Usually	10%	0%
**2.** How often do you see **halos**?	Never	60%	40%
	Occasionally	30%	40%
	Often	0%	20%
	Usually	10%	0%
**3.** How often do you see **flashes**?	Never	40%	50%
	Occasionally	20%	30%
	Often	0%	10%
	Usually	40%	10%
**4.** How often do you have the feeling of looking through **dirty glass**?	Never	60%	90%
	Occasionally	40%	10%
	Often	0%	0%
	Usually	0%	0%
**5.** How often do you have **blur vision**?	Never	60%	90%
	Occasionally	40%	10%
	Often	0%	0%
	Usually	0%	0%
**6.** How often do you have **distortion vision**?	Never	70%	90%
	Occasionally	30%	10%
	Often	0%	0%
	Usually	0%	0%
**7.** How often do you have **double vision**?	Never	80%	90%
	Occasionally	20%	10%
	Often	0%	0%
	Usually	0%	0%
**8.** How often do you have **fluctuations in your vision**?	Never	60%	70%
	Occasionally	30%	30%
	Often	10%	0%
	Usually	0%	0%
**9.** How often do you have **difficult to focus**?	Never	40%	40%
	Occasionally	50%	50%
	Often	10%	0%
	Usually	0%	10%
**10.** How often do you have difficult to **perceive distances** or **depth**?	Never	80%	80%
	Occasionally	20%	20%
	Often	0%	0%
	Usually	0%	0%

#### Satisfaction questionnaire

Table 5 shows the subjective satisfaction reported by patients at 12 months postoperatively. The satisfaction questionnaire shows that overall, 90% of patients were satisfied with their visual results: 80% of patients were very satisfied, 10% of patients were quite satisfied with the surgery, and 10% of patients were dissatisfied with their visual outcomes.

**Table 5:** Outcomes of satisfaction questionnaire (12 months postop)

**1.** How satisfied are you with the overall result of the procedure?			
Dissatisfied 10%	A little 0%	Quite satisfied 10%	Very satisfied 80%
**2.** How satisfied are you with your uncorrected vision?			
No 10%	A little 0%	Considerably 40%	A lot 50%
**3.** How satisfied are you with your near vision?			
No 10%	A little 20%	Considerably 10%	A lot 60%
**4.** How satisfied are you with your intermediate vision?			
No 10%	A little 20%	Considerably 50%	A lot 40%
**5.** How satisfied are you with your far vision?			
No 0%	A little 10%	Considerably 40%	A lot 50%
**6.** Did you get the visual quality you wanted?			
No 10%	Maybe 0%	Considerably 10%	Ýes 80%
**7.** Has this lens been a good choice?			
No 10%	A little 0%	Considerably 0%	A lot 50%
**8.** With your current experience, would you operate again?			
No 10%	Maybe 0%	Considerably 0%	Ýes 90%
**9.** Would you recommend the implantation of this lens to another person?			
No 10%	Maybe 0%	Considerably 0%	Ýes 90%
**10.** How often do you still wear your glasses or contact lenses?			
Never 60%	Occasionally 20%	Often 20%	Most often 0%
**11.** If you wear glasses, for what distance?			
Not available 60%	Near vision 40%	Intermediate vision 0%	Far vision 0%

Sixty percent of patients answered that they had not required the use of glasses after surgery, 20% had used spectacles for near vision “occasionally” and the other 20% had “often” used spectacles for near vision.

## Discussion

Multifocal IOLs aim to provide a good distance, intermediate and near visual outcomes because more often, daily activities requires a high range of vision (for example, use of computers, tablets or car dashboard) [1, 2].

In agreement with previous studies with multifocal IOLs (better than 0.10 logMAR) [1], this IOL provides a good VA (0.08 ± 0.08 logMAR) for distance vision [2, 11–30]. Near vision outcomes achieved in this study (0.22 ± 0.12 logMAR) were compatible with other publications (better than 0.30 logMAR) with difference models of multifocal IOLs [1, 2, 11, 13, 14, 16, 17, 19–21, 24, 26–33]. The intermediate visual outcomes obtained in this study (0.22 ± 0.10 logMAR) were similar with other reports (better than 0.30 logMAR) [1, 2, 12, 13, 16, 17, 21, 22, 24, 25, 31–33].

The outcomes of the defocus curve binocular analysis show that this IOL provides an acceptable VA with a defocus range of + 1.00 to − 2.50 D (VA equal or better to 0.16 LogMAR), according to a previous study which defined 0.30 logMAR as the limit of good vision [9]. The outcomes of the defocus curve show good values of VA for far vision defocus (− 0.08 ± 0.08 logMAR), near defocus (0.16 ± 0.15 logMAR) and intermediate defocus (0.12 ± 0.11 logMAR). Outcomes in binocular defocus curve are similar to other IOLs [10, 34].

In this study, contrast sensitivity was measured with the CSV-1000 test in different conditions of light, photopic and mesopic conditions and with and without glare in binocular vision. Regarding contrast sensitivity outcomes, this multifocal IOL provides contrast sensitivity values similar to the normal population, for an age group between 50 and 75 years of age, in photopic lighting conditions and slightly reduced in mesopic conditions [35]. Another previous study reported lower contrast sensitivity in multifocal IOLs in mesopic lighting conditions [36].

The subjective quality of vision and patient’s satisfaction was evaluated with two questionnaires at 3 and 12 months after surgery. Outcomes in quality of vision questionnaire at 12 months show that a high percentage of patients reported that they had never seen glares (70%) and 80% of patients reported that they “never” or only “occasionally” seen halos (40% “never”; 40% “occasionally”), and 20% of patients reported that they had seen halos “often” and 0% of patients reported “usually” seeing halos.

The satisfaction questionnaire revealed that 90% of patients were satisfied in general with their visual results and 10% of patient reported that they were dissatisfied with visual outcomes. Only one patient reported was dissatisfied, the reason was a poor neuroadaptation and discomfort due to perception of photic phenomena, especially at night, and the fact that he was a demanding patient, despite having a good VA and not showing a postoperative refractive defect. 60% of patients answered that they had not required the use of glasses after surgery, 20% had used spectacles for near vision “occasionally” to perform near visual activities which require precise details or read small print letters and 20% of patient had used spectacles for near vision “often”.

The main limitation of the current work is its pilot nature, which includes only a several cases [37]. Recently, another study reporting clinical outcomes with this lens has been published [5]. In that study, Royo et al. [5] included both eyes for analysis of VA and refractive parameters. Regarding VA values, results were obtained 6 months after the operation, while in our study results were obtained at 12 months. The monocular and binocular UDVA values were similar (0.03 logMAR and 0.01 logMAR, respectively). The UNVA and UIVA values were slightly higher than those obtained in this study. This may be due to the value of SE mean (− 0.54 D) being slightly more myopic than our research. These previous results can be reflected by comparing the defocus curve; where it can be observed that our curve is displaced towards the top in far defocus, while we observed similar outcomes for intermediate vision, and our outcomes for near defocus were slightly reduced. In general, their defocus curve outcomes, same as our results, provides an acceptable VA with a defocus range of + 1.00 to − 2.50 D (0.30 logMAR as the limit of good vision) [9]. Values of contrast sensitivity and photic phenomena were similar. Nevertheless, further studies with larger samples and without this potential bias are required for confirming these outcomes. The difference compared with Royo et al. [5] study, is that they asked verbally about the perception of disturbing photic phenomena while in our study we measured subjectively the perception of photic phenomena through a validated questionnaire [7].

## Conclusions

In conclusion, the Precizon Presbyopic NVA IOL provides good visual outcomes. This multifocal IOL provides a high percentage of spectacle independence after bilateral implantation. Patients reported satisfactory full range of vision with good values of VA at far, intermediate and near distances. Patients obtained good contrast sensitivity. Good patient satisfaction was shown in quality of vision and satisfaction questionnaires with a low percentage of patients manifesting photic phenomena. Future investigations are required to determine the long follow-up outcomes with this new multifocal IOL.

## Data Availability

The datasets used and/or analysed during the current study are available from the corresponding author on reasonable request.
